# Identification of Darunavir Derivatives for Inhibition of SARS-CoV-2 3CL^pro^

**DOI:** 10.3390/ijms232416011

**Published:** 2022-12-16

**Authors:** Ling Ma, Yongli Xie, Mei Zhu, Dongrong Yi, Jianyuan Zhao, Saisai Guo, Yongxin Zhang, Jing Wang, Quanjie Li, Yucheng Wang, Shan Cen

**Affiliations:** Institute of Medicinal Biotechnology, Chinese Academy of Medical Science, Beijing 100050, China

**Keywords:** severe acute respiratory syndrome coronavirus 2, 3CL^pro^, anti-coronavirus drug, screening assay, protease

## Abstract

The effective antiviral agents that treat severe acute respiratory syndrome coronavirus 2 (SARS-CoV-2) are urgently needed around the world. The 3C-like protease (3CL^pro^) of SARS-CoV-2 plays a pivotal role in virus replication; it also has become an important therapeutic target for the infection of SARS-CoV-2. In this work, we have identified Darunavir derivatives that inhibit the 3CL^pro^ through a high-throughput screening method based on a fluorescence resonance energy transfer (FRET) assay in vitro. We found that the compounds 29# and 50# containing polyphenol and caffeine derivatives as the P2 ligand, respectively, exhibited favorable anti-3CL^pro^ potency with EC50 values of 6.3 μM and 3.5 μM and were shown to bind to SARS-CoV-2 3CL^pro^ in vitro. Moreover, we analyzed the binding mode of the DRV in the 3CL^pro^ through molecular docking. Importantly, 29# and 50# exhibited a similar activity against the protease in Omicron variants. The inhibitory effect of compounds 29# and 50# on the SARS-CoV-2 3CL^pro^ warrants that they are worth being the template to design functionally improved inhibitors for the treatment of COVID-19.

## 1. Introduction

The pandemic of COVID-19 caused by infection with SARS-CoV-2 has had an unprecedented impact on the health and economic development of the world, which has led to over 611 million cumulative cases as of 23 September 2022 (Coronavirus disease (COVID-19) (who.int)). SARS-CoV-2 is a positive-sense enveloped RNA virus with a single-strand, which shares about 80% of its RNA genome identity with SARS-CoV of the genus Betacoronaviru [1,2]. According to the World Health Organization (WHO), there are no clinically effective vaccines and targeted therapeutics for SARS-CoV-2 infections. Up to now, although several medications are currently being evaluated in clinical trials, and some kinds of vaccines have been used to prevent the occurrence of SARS-CoV-2, it will take a long time to effectively cure patients or vaccinate the world population faced with viral mutants. Therefore, effective and curative treatment measures are still urgently needed for COVID-19.

SARS-CoV-2 is an RNA virus that is ~30,000 nt in length. Its two overlapping open reading frames (ORF)1a and ORF1b, located at two-thirds of the viral genome from the 5′ terminus, encode two polyproteins, pp1a and pp1ab [1,3]. These replicase polyproteins will be cleaved by two different proteins, the papain-like cysteine protease (PL^pro^) and 3CL^pro^, encoded in the ORF1a to generate 16 nonstructural proteins (NSPs) for viral genome transcription and replication [4]. The 3CL^pro^, also referred to as the main protease (M^pro^), plays an important role in the maturation of SARS-CoV-2, and the inhibition of the activity of the 3CL^pro^ is expected to block the viral replication. Meanwhile, the sequence alignment shows that SARS-CoV-2 3CL^pro^ is about 96% and 50% identical to the M^pro^ of SARS-CoV and MERS-CoV, respectively, which also reveals a structural similarity of the 3CL^pro^ among the three CoVs [1]. The 3CL^pro^ cleaves 11 out of 14 of the conserved sites, and it prefers to recognize the substrates involving the Leu-Gln ≤ ↓ (Ser, Ala, Gly) sequence; additionally, there are no known human proteases with a similar cleavage specificity [5,6]. Overall, the conservation features of the 3CL^pro^ and its functional importance in the viral life cycle have turned it into an attractive antiviral target for the development of broad-spectrum, less toxic, and effective anti-CoVs drugs.

Several crystal structures of the unliganded SARS-CoV-2 3CLpro and its complex with inhibitors have been resolved [7,8,9]. The studies have suggested that these compounds, N3 and ebselen [8], 11a and 11b [9], and improved α-ketoamide inhibitors [7], show the potential to develop as promising drug candidates against COVID-19. The availability of the crystal structure of SARS-CoV-2 3CL^pro^ also promotes many initial computational studies to screen for compounds or existing drugs against COVID-19 [10,11,12,13,14,15]. At present, several studies have been reported to identify the potential inhibitors targeting the 3CL^pro^ of SARS-CoV-2. The oral SARS-CoV-2 3CL^pro^ inhibitor nirmatrelvir has been approved by the FDA in combined use with ritonavir (Paxlovid) for Covid 19 treatment [16,17,18]. The study of the molecular complexation between each inhibitor and SARS-CoV-2 3CL^pro^ indicated that both drugs interact well with the residues at the active site of SARS-CoV-2 3CL^pro^ [19]. It is reported that several HIV-1 protease inhibitors (HIV-1 PIs) approved by the FDA are active against SARS-CoV-2 and may have demonstrated antiviral activity towards the 3-chymotrypsin-like protease (3CL^pro^) in the emergency phase of the COVID-19 pandemic. Darunavir, a protease inhibitor used in HIV therapy [20,21], with cobicistat as a pharmaco-enhancer, has also been proposed as a COVID-19 treatment [22]. However, evidence for the efficacy of DRV/c in COVID-19 patients is scarce. Inspired by the above, we test several kinds of HIV-1 protease inhibitors, which were designed and synthesized in our laboratory in this study, and found that Darunavir derivatives 29# and 50# containing polyphenol and caffeine derivative as the P2 ligand, respectively, exhibited favorable anti-SARS-CoV-2 potency with IC50 values of 6.3 and 3.5 μM in vitro, as well as EC50 values of 8.9 and 13.5 μM at the cellular level, respectively. Meanwhile, 29# and 50# have been identified and showed high-binding affinities to SARS-CoV-2 3CL^pro^, which further suggested their strong potential to develop as COVID-19 drugs. Our results suggest that compounds 29# and 50# are worthy to act as hit compounds that will further improve functionally for the treatment of the SARS-CoV-2 infection.

## 2. Results

### 2.1. Establishing a High-Throughput Screening System with FRET Assay Targeting SARS-CoV-2 3CL^pro^

**Figure 1:**
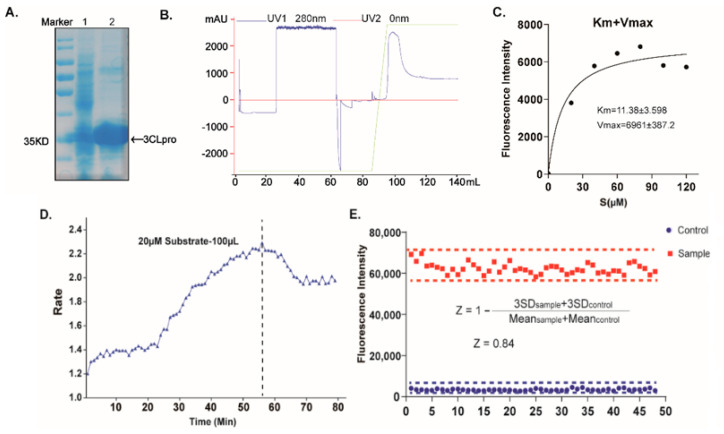
Establishing a high-throughput screening system with FRET assay. (**A**) SDS–PAGE analysis of the 3CL^pro^. Lane 1 represents the molecular mass markers, 35 kDa. Lane 2 shows the cell lysate, and lane 3 shows the 3CL^pro^ with the tag, respectively. (**B**) Purification flow-through curve. (**C**) Measurements of kinetic parameters of the 3CL^pro^. (**D**) Dependence of the 3CL^pro^ reaction rate on the reaction time. (**E**) Summary of statistical parameters to assess the robustness of the HTS assay.

To identify candidate inhibitors accurately and quickly, we first established a high-throughput screening system with a FRET assay targeting SARS-CoV-2 3CL^pro^ in vitro [9,23]. The full-length gene encoding SARS-CoV-2 3CL^pro^ was codon-optimized and inserted into the pET-28a^(+)^ vector, whereafter it was transformed into the BL21(DE3) Escherichia Coli strain and high-yieldingly expressed with a His-tag in its C-terminus. The recombinant 3CL^pro^ was purified with a Ni-NTA column to high purity (Figure 1). The enzymatic activity of the 3CL^pro^ was then evaluated by a fluorescently labeled peptide substrate, MCA-AVLQ↓SGFR-Lys(Dnp)-Lys-NH2, based on the auto-cleavage sequence of the 3CL^pro^ N-terminal. Additionally, the FRET assay had an obvious difference between the negative group (substrate + inactivating 3CL^pro^) and workgroup (substrate + activating 3CL^pro^). The assay was further optimized for high-throughput screening by the assessment of its statistical parameters, as previously described [24], to determine the performance and robustness of this assay. The results showed that all the Z factors (0.84), %CV (13%), and S/N(22.3), met the criteria required for the high-throughput screening (HTS) assay (Figure 1). Together, we successfully established a high-sensitivity and high-throughput screening system with the FRET assay to find the inhibitors targeting the recombinant 3CL^pro^.

### 2.2. Screening of DRV Derivatives against SARS-CoV-2 3CL^pro^

Using the high-throughput FRET assay, we screened a collection of DRV derivatives to identify potential 3CL^pro^ inhibitors (Table 1). These DRV derivatives are grouped based on their structure, including pentacyclic triterpenoids [25], purine or pyrimidine bases [26,27], morpholines [28], piperidines, coumarin derivatives [29], phenols or polyphenols [30,31], caffeine derivatives, and so on.

**Figure 2:**
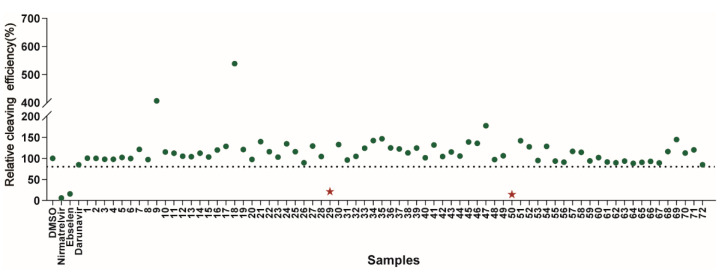
Results of the high-throughput screening. Twenty µM of the compounds was pre-incubated with 100 nM of SARS-CoV-2 3CL^pro^ for 30 min at 30 °C, and then a 10 µM FRET substrate was added to the reaction mixture to initiate the reaction. The reaction was monitored for 2 h. The initial velocity was calculated by linear regression using the data points from the first 15 min of the reaction. The calculated initial velocity with each compound was normalized to DMSO control. The results are the average of three repeats. The asterisk indicates 29# contains a polyphenol P2 ligand, and 50# contains a caffeine P2 ligand.

The primary screening of these derivatives was conducted at a single concentration of 20 μM. We used nirmatrelvir, ebselen, and darunavir as comparisons, respectively, and DMSO-treated wells served as an intraplate negative control for the data normalization [8]. At least triplicate repeats were performed for each tested compound and the control. The results show that nirmatrelvir and ebselen can strongly inhibit 3CL^pro^ activity, as reported(Figure 2). The DRV exhibited no inhibitory activity using this assay, which is consistent with the efficacy of the DRV/c in clinics [22]. The initial hits were defined as those active compounds that displayed cleaving efficiency with a significant difference compared with that of the control group, which identified two compounds (29# and 50#) with significant inhibition against SARS-CoV-2 3CL^pro^. Among the hits, 29# contains a polyphenol P2 ligand, and 50# contains a caffeine P2 ligand.

### 2.3. The Inhibitory Activity of Hits against SARS-CoV-2 3CL^pro^ In Vitro

**Figure 3:**
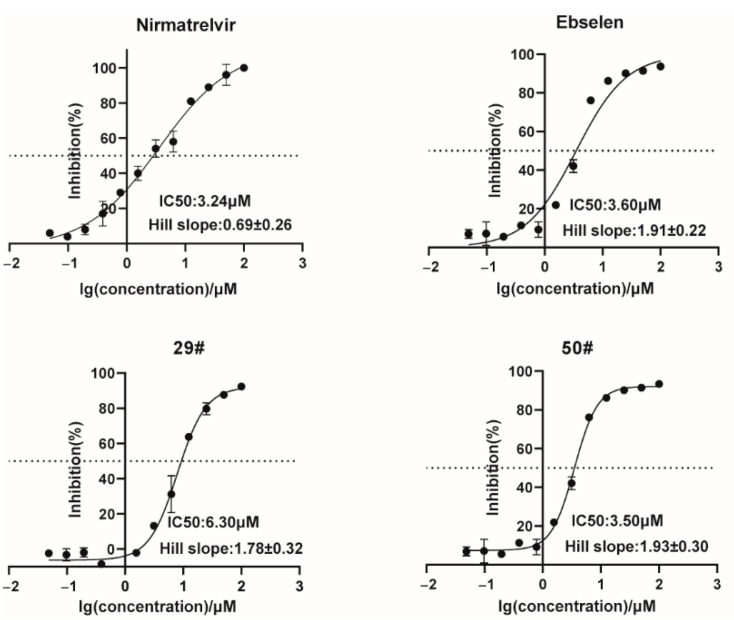
Dose-response inhibition curves of selected hits against the 3CL^pro^. The hits with different concentrations were pre-incubated with 100 nM of SARS-CoV-2 3CL^pro^ for 30 min at 30 °C, and then a 10 µM FRET substrate was added to the reaction mixture to initiate the reaction. The reaction was monitored for 2 h. The initial velocity was calculated by linear regression using the data points from the first 15 min of the reaction. The calculated initial velocity with each compound was normalized to the DMSO control. The results are an average ±standard deviation of three repeats.

To verify the screening results, we further characterized the inhibitory activities of these hits against SARS-CoV-2 3CL^pro^ in a dose-response experiment to determine their IC_50_ values. The FRET values were measured, and the inhibitory rates were calculated for the inhibitor treatments compared to the DMSO control, which was then plotted as dose-response curves for determining the inhibitor efficacies of the IC_50_ values. The results showed that nirmatrelvir and ebselen could dose-dependently inhibit the enzymatic activity of the 3CL^pro^ with an IC_50_ value of 3.24 μM and 3.60 μM (Figure 3), which are comparable to the reported IC_50_ value. We also observed a gradual decrease in the cleavage efficacy of the 3CL^pro^ under the treatment of our hits (29# and 50#) with an IC_50_ value of 6.30 μM and 3.50 μM, respectively. As a counter screen, we measured the activity of 29# and 50# against Cathepsin L, a member of the lysosomal cysteine protease [32]. The result showed that both the compounds exhibited a very limited inhibitory effect (Figure S1), providing evidence to support the specific activity of 29# and 50# against the 3CL^pro^.

### 2.4. Binding of Hits to SARS-CoV-2 3CL^pro^

We next performed a bio-layer interferometry (BLI) kinetic binding assay to assess the binding capacity of these hits to SARS-CoV-2 3CL^pro^ in vitro in expectation of exploring the possible mechanisms of these hits against SARS-CoV-2 3CL^pro^. The purified SARS-CoV-2 3CL^pro^ was immobilized on the biosensor surface and then incubated with different concentrations of the hits. Meanwhile, a binding assay tested the fitting association and dissociation response curves of the inhibitors after different concentration hits and made a blank control containing only the sample buffer for reference.

**Figure 4:**
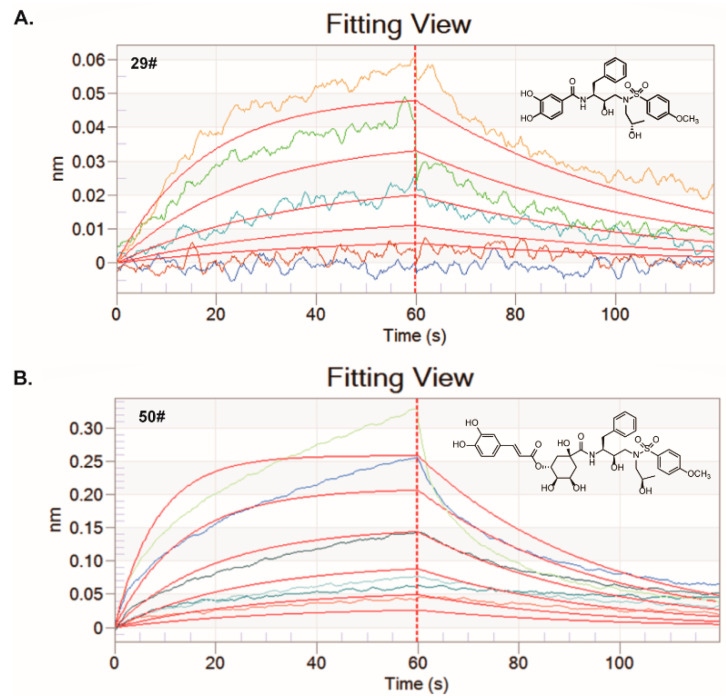
BLI assays for the binding of the hits to the 3CL^pro^ protein in vitro. Both of the hits at different serially diluted concentrations were titrated into a fixed concentration of the labeled 3CL^pro^ protein (100 nM). (**A**) The kinetic fitting curves for the interaction between 29# and 3CL^pro^ protein was analyzed by BLI assay. Five different concentrations including 6.25, 12.5, 25, 50, and 100 µM were set. (**B**) The kinetic fitting curves for the interaction between 50# and 3CL^pro^ protein was analyzed by BLI assay. Six different concentrations including 6.25, 12.5, 25, 50, 100 and 200 µM were set.

The BLI results revealed that 29# and 50# bind to SARS-CoV-2 3CL^pro^ in a dose-dependent manner (Figure 4 and Table 2). The kinetic affinity constant values (K_D_) of the hits were determined. 29# and 50# exhibited high affinity with the K_D_ values of 52 μM and 62 μM, respectively. These results further support and explain the indirect function of the nominated hits that were provided by the enzymatic inhibition assay in vitro on the anti-SARS-CoV-2 3CL^pro^ activity. To rule out the possibility of non-specific binding, we measured the binding affinity of 29# and 50# with the Norovirus RNA-dependent RNA polymerase (RdRp) using the BLI assay (Figure S2). The results showed either a large K_D_ value or a not-detected K_D_ value, suggesting the specific binding of 29# and 50# to SARS-CoV-2 3CL^pro^.

### 2.5. Modes of Interaction for SARS-CoV-2 3CL^pro^ with Hits

**Figure 5:**
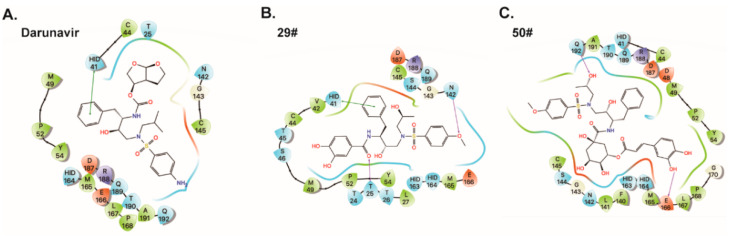
Predicted binding poses for darunavir (**A**) and its two derivatives (**B**,**C**). Residues within 5 Å of the compounds were shown on the 2D ligand interaction diagram. For clarity, only polar hydrogen atoms were shown. Here, the color code is that dark blue is positively charged, red is negatively charged, cyan is polar, and green is hydrophobic. Hydrogen bonding is depicted as the magenta arrows, π-cation interactions are depicted as the red line, and π–π interactions are depicted as the green line.

To explore the binding mode of the identified inhibitors, molecular docking studies of the DRV and its two derivatives (29# and 50#) against SARS-CoV-2 3CL^pro^ were performed by AutoDock Vina. The calculated binding energies (DG_ADV_) of the DRV, 29#, and 50# were −7.1 kcal/mol, −7.8, and −7.6 kcal/mol, respectively. Due to their common structural skeleton, all the compounds presented a similar arrangement within the binding cavity of SARS-CoV-2 3CL^pro^ (Figure 5). As shown in Figure 5A, the binding pocket of the DRV is located in the catalytic cavity of SARS-CoV-2 3CL^pro^. The benzene ring of the DRV formed a π–π interaction with H41, one of the catalytic dyad residues that are important for the proteolytic activity of SARS-CoV-2 3CL^pro^. Compared with the DRV, 29# formed two additional H-bonds with the 3CL^pro^ active site residues, T25 and N142 (Figure 5B), respectively. As for inhibitor 50#, after the introduction of the *o*-dihydroxybenzene group (Figure 5C), 50# formed a H-bond with E166, a key residue for the dimerization of the 3CL^pro^ [8]. Additionally, another H-bond interaction was observed between 50# and the active site residue, Q192. This may account for the stronger inhibitory activity of DRV derivatives 29# and 50#. Taken together, the data suggest a direct interaction between the hits and SARS-CoV-2 3CL^pro^.

In addition, in the predicted binding poses of compounds 50# and 29#, the phenolic hydroxyl groups at the P2 position formed H-bonds with the 3CL^pro^ active site residues, such as N142, T25, and E66. This phenolic hydroxyl group may be largely absent in those series of compounds that show no activity against SARS-CoV-2 3CL^pro^. Thus, we speculated that the introduction of the phenolic hydroxyl group at the P2 position may improve the inhibitory effect of the compounds.

### 2.6. The Inhibitory Activity of Hits against SARS-CoV-2 3CL^pro^ at Cellular Level

**Figure 6:**
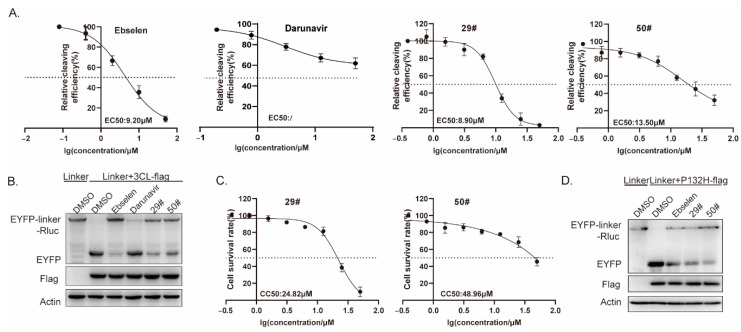
Effect of hits on 3CL Omicron variants. (**A**) HEK293T cells were co-transfected with the 3CL-flag and EYFP-linker-Rluc plasmid DNA. At 12 h post-transfection, the cells were re-seeded in 96-well plates (10^4^/well) and treated with the serially diluted hits. After 36 h, the BRET ratio was measured. (**B**,**D**) HEK293T cells (4 × 10^5^) were co-transfected with the 3CL-flag(or P132H-flag) and EYFP-linker-Rluc plasmid DNA. At 12 h post-transfection, the cells were treated with the hits (20 μM). After 48 h post-transfection, cells were collected for a Western blot. (**C**) CC_50_ values in the cells were measured using CCK-8 kits. All data are expressed as the mean ± SD of the triplicate assays.

To further confirm and characterize the inhibitory activities of these hits against SARS-CoV-2 3CL^pro^ at the cellular level, we performed a cell-based bioluminescence resonance energy transfer (BRET) assay, as previously described [33]. HEK293T cells were transfected with plasmids expressing pEYFP-linker-Rluc and SARS-CoV-2 3CL^pro^ before being treated with increasing doses of the inhibitors. The BRET values were then measured, and the inhibitory rates were calculated for the inhibitor treatments compared to the DMSO control, which was then plotted as dose-response curves for determining the inhibitor efficacies of the EC_50_ values (Figure 6A). From the results, the EC_50_ values of the two hits ranged from 8.9 to 13.5 μM, with the positive control, ebselen, at 9.2 μM. Meanwhile, we also evaluated the cytotoxicity of the inhibitor hits using a Cell Counting Kit-8 (CCK-8) assay. The results show that the CC_50_ values of the two hits are higher than 24 μM (Figure 6C), which generally rules out the false positives due to significant cell toxicity. Ebselen has extremely low cytotoxicity (the median lethal dose in rats is >4600 mg kg^−1^ when taken orally), and darunavir is the latest protease inhibitor approved by the FDA; both are safe in humans and have been evaluated in a number of clinical trials [7,34]. It should be noted that the relatively low selectivity indexes of 29# and 50# suggest that their activity might be in part due to cytotoxicity; therefore, further optimization is clearly needed for future development.

Next, we explored a mutation, P132H, in the 3CL^pro^, which was included in the SARS-CoV-2 Omicron variant. Based on the predicted binding model, this mutation most unlikely affected the binding of the two hits to the 3CL^pro^; therefore, no difference in the susceptibility was expected. Whereafter, we performed a Western blot assay to validate the inhibition of the two hits on the SARS-CoV-2 3CL^pro^ enzymatic activity. In line with it, we found a similar effect of the hits on the cleavage efficiency of the 3CL^pro^ containing the mutation P132H (Figure 6D).

## 3. Discussion

The Coronaviruses such as SARS, MERS, and SARS-CoV-2 that broke out in 2019 have caused three large-scale pandemics in human history during the past 20 years. With the ongoing outbreak of SARS-CoV-2, scientists and researchers around the world are facing finding effective antiviral drugs or vaccines with different strategies in a short time [35]. One of the quickest and relatively safest ways to find therapeutic drugs is to repurpose the clinical drug. Remdesivir, the viral polymerase inhibitor, shows the greatest treatment effect and is being evaluated in several clinical trials [36], and it also obtained emergency-use authorization from the FDA. Lopinavir and Ritonavir, which are HIV drugs, were also reported in some research. However, the combination of the two HIV drugs failed in a clinical trial for the SARS-CoV-2 infection, as no significant therapeutic efficacy was observed [37].

To address this urgent medical need, we designed and initiated a drug repurposing screening to identify effective inhibitors against SARS-CoV-2 3CL^pro^ from the structurally improved protease inhibitors that were approved by the FDA. The M^pro^ has been shown to be a validated antiviral drug target for SARS-CoV and MERS-CoV [38]. As SARS-CoV-2 3CL^pro^ (also referred to as M^pro^) shares high sequence similarity with SARS-CoV and, to a lesser extent, with MERS-CoV, studies have also reasoned that inhibiting the enzymatic activity of SARS-CoV-2 3CL^pro^ will similarly prevent viral replication [6]. Herein, we designed and synthesized a series of compounds improved from several kinds of HIV-1 protease inhibitors that had been used in clinical. Through testing with a high-throughput screening system with a recombinational 3CL^pro^, we found that compounds 29# and 50# exhibited favorable anti-SARS-CoV-2 3CL^pro^ potency, with IC_50_ values of 6.30 and 3.50 μM and EC_50_ values of 8.90 and 13.50 μM. Therefore, we successfully designed two hits that are worthy of being functionally improved for the treatment of COVID-19.

The known SARS-CoV-2 3CL^pro^ inhibitors in the existing studies mainly include two types: peptide inhibitors and small molecule compounds. The reported small molecule inhibitors targeting the 3CL^pro^ are significantly less than the peptide inhibitors, possibly because the latter are designed on the peptidomimetic substrates with known and various chemical warheads, such as α-ketoamide, aldehyde, aldehyde prodrug bisulfite, chloromethyl ketones, and others [6]. Herein, we designed compounds based on these chemical warheads. The 29# and 50# compounds have similar activity to the recently reported SARS-CoV-2 3CL^pro^ inhibitors (ebselen, N3, and 13b), which also represent the potent and selective peptidomimetic hits designed and renovated based on the chemical warheads.

In summary, this study identified two potent SARS-CoV-2 3CL^pro^ inhibitors with convincing enzymatic inhibition in vitro, as well as cellular antiviral activity. Further improvement based on the structure of these hits might produce clinically therapeutic drugs for SARS-CoV-2 infection.

## 4. Materials and Methods

### 4.1. Cell Culture and Transfection

HEK293T cells were maintained in Dulbecco’s modified Eagle’s medium (DMEM, Gibco, Grand Island, NY, USA) supplemented with 10% fetal bovine serum (FBS, Gibco, Grand Island, NY, USA) and a 1% penicillin-streptomycin solution (Invitrogen, Carlsbad, CA, USA) at 37 °C and 5% CO_2_. For cell transfection, cells were plated at a density of 2 × 10^5^ cells/mL. After 24 h incubation, cells were transfected using Lipofectamine 2000 (Invitrogen, Carlsbad, CA, USA), according to the manufacturer’s instructions.

### 4.2. Plasmid, Antibodies, and Reagents

The pEYFP-linker-Rluc expression construct was generated by PCR using pEYFP-N1 (Clontech, San Jose, CA, USA) as a template, with the designed primers harboring the 3CL^pro^ recognition sequence (linker: ITSAVLQSGFRK). The PCR-amplified EYFP-linker fragment was then inserted into a pRLuc-N2 expression construct (PerkinElmer, Waltham, MA, USA). The full-length gene encoding SARS-CoV-2 3CL^pro^ was codon-optimized and synthesized (Tsingke Biotechnology Co., Ltd., Beijing, China) and subcloned into a pcDNA3.1 vector with a C-terminal FLAG-tag to produce the pcDNA3.1-3CL^pro^-FLAG expression plasmid.

Coelenterazine-h was purchased from Promega. Compounds were designed and synthesized in our laboratory and prepared in DMSO. The DYKDDDDK (Flag) Tag antibody (8146) was purchased from Cell Signaling Technology, Inc (CST, Danvers, MA, USA), and the β-actin antibody (ab8224, Cambridge, UK) was purchased from Abcam.

### 4.3. In Vitro Enzymatic Inhibition Assay

The enzyme activity and inhibition assays have been described previously [9]. The recombinant SARS-CoV-2 3CL^pro^ (1 μM at the final concentration) was mixed with serial dilutions of each compound in a 50 µL assay buffer (50 mM Tris-HCl, pH 7.3, 1 mM EDTA) and incubated for 5 min. The reaction was initiated by adding a 50 µL fluorogenic substrate with a final concentration of 20 µM and then incubated for 30 min with gentle shaking at room temperature. After that, the fluorescence signal at 320 nm (excitation)/405 nm (emission) was measured every 5 min and 10 times with a PerkinElmer plate reader. The Vmax of the reactions added with the compounds at various concentrations compared to the reaction added with the DMSO was calculated and used to generate IC50 curves. For each compound, IC50 values against SARS-CoV-2 3CL^pro^ were measured at nine concentrations, and three independent experiments were performed. All experimental data were analyzed using GraphPad Prism software(San Diego, CA, USA).

### 4.4. BRET Assay

For the BRET assay, transfected cells were seeded in 96-well white plates with a clear bottom. After overnight incubation, the DMSO (Sigma-Aldrich, Burlington, MA, USA) or compounds were added to each well at the designated concentration. After further incubation for 48 h at 37 °C, the supernatant was discarded. A stock of coelenterazine-h was dissolved in absolute ethyl alcohol to a concentration of 1.022 mmol/L. For the luminescence assay, 100 μL of 10 μM coelenterazine-h was injected, and the luminescence was measured using a VICTOR^TM^ X5 2030 Multilabel Reader (PerkinElmer, USA).

### 4.5. Western Blot

HEK293T cells were seeded at a density of 2 × 10^5^ cells per well in a 6-well plate. After overnight incubation, the DMSO (Sigma-Aldrich) or compounds were added to each well at the designated concentration. After further incubation for 48 h at 37 °C, transfected HEK293T cells were collected and lysed in a RIPA buffer containing 50 mM of Tris (pH 7.4), 150 mM of NaCl, 1% NP-40, 0.1% SDS, 1 mM of EDTA, and 0.5% sodium deoxycholate. Cell lysates were mixed with an SDS-loading buffer and boiled for 20 min, followed by electrophoresis in 10% polyacrylamide-SDS gels. Proteins were transferred onto the PVDF membrane. After blocking with 5% skim milk, the membrane was blotted with anti-flag (1:1000) or anti-β-actin (1:5000) antibodies. After incubation with HRP-conjugated secondary antibodies (1:5000), protein signals were detected with enhanced chemiluminescence.

### 4.6. Cell Viability Assay

Cell viability was performed by a Cell Counting Kit-8 (CCK-8) (Beyotime, China). Typically, HEK293T cells were seeded in the wells of a 96-well plate at 100 μL/well and grown for 24 h. Then, 10 μL of CCk-8 reagent and incubated for 1.5 h at 37 °C with 5% CO_2_. The absorbance at 450 nm was subsequently measured using an EnSpire 2300 Multilabel Reader (PerkinElmer).

### 4.7. Bio-Layer Interferometry (BLI) Binding Assay

All binding affinity and kinetic profiles were conducted using a ForteBio Octet RED96 instrument (ForteBio, Inc., CA, USA) equipped with super streptavidin biosensor chips (ForteBio). Purified His-tagged nsp12 (50 μg/mL) were captured via Ni-NTA biosensors (960 s, at 25 °C, with 1000 rpm). Ligand biosensors and reference biosensors were dipped into multiple concentrations of the test compounds for 60 s (kon,1/Ms), then dissociated for 60 s (kdis,1/s). The blank binding using a buffer was used to correct the baseline shift during the analysis. Data analysis on the ForteBio Octet RED instrument was performed using reference well subtraction in the ForteBio data analysis software.

### 4.8. Docking (or Modeling)

To explore the binding mode of the identified inhibitors, a molecular docking study of simeprevir against SARS-CoV-2 3CL^pro^ (PDB-ID: 6M2N) was performed by AutoDock Vina. Protein structures were prepared for docking using AutoDockTools (ADT, version: 1.5.6) by removing co-crystalized water molecules, adding polar hydrogens, and merging Gasteiger charges. The active site of the 3CL^pro^ was defined as a box of 22 Å × 22 Å × 22 Å, centered near the ligand binding region (x = −32.169 Å, y = −64.8 Å, z = 41.176 Å). The grid step size for each docking volume was set to 1 Å. Ligands of the DRV, 29#, and 50# were prepared using an Open Babel toolkit version. Auto Dock Vina software package was used for docking, as previously described [39].

### 4.9. Quantification and Statistical Analysis

Data are presented as the mean ± standard deviation (SD) from at least three independent experiments unless otherwise indicated and were analyzed with GraphPad Prism.

## Figures and Tables

**Table 1:**
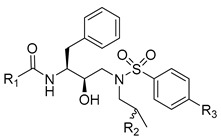
HIV-1 protease inhibitors with different types in the P2 ligand.

Type in P2 Ligands	R_1_		R_2_	R_3_
**Pentacyclic triterpenoid**	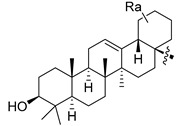	R_a_ = H, or CH_3_	CH_3_	OCH_3_, NO_2_, or NH_2_
**Purine**	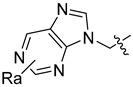	R_a_ = OCH_3_, H, Cl or NH_2_	CH_3_	OCH_3_, NO_2_, or NH_2_
**Pyrimidine**	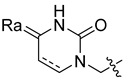	R_a_ = O, 2H, or NH_2_	CH_3_	OCH_3_, NO_2_, or NH_2_
**Morpholine**	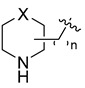	X = O, or SO_2_; n = 1, or 2	CH_3_	OCH_3_, NH_2_, NHOH, NO_2_, or CF_3_
**Piperidine**	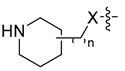	X = CH_2_, or O; n = 0, or 1	CH_3_	OCH_3_, or NH_2_
**Coumarin**	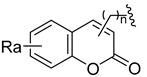	R_a_ = H, CH_3_, NH_2_, OH, or OCH_3_	CH_3_	OCH_3_, NO_2_, or NH_2_
**Phenol**	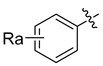	R_a_ = H, OCH_3_, OH, or Cl	CH_3_, (*R*)-OH, or (*S*)-OH	OCH_3_, NO_2_, NH_2_, F, or CF_3_
**Phenylpropionic acid**	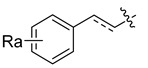	R_a_ = H, OCH_3_, or OH	H, or CH_3_	OCH_3_, SCH_3_, or NH_2_

**Table 2:** Kinetics of 29# and 50# binding to the 3CL^pro^ in the ForteBio’s Octet System.

Analyte	Ligand (3CL^pro^)					
	K_on_^*^ (1/Ms)	K_on_^*^ Error	K_dis_^*^ (1/s)	K_dis_^*^ Error	K_D_^*^ (M)	K_D_^*^ Error
29#	3.74 × 10^2^	16.3	1.97 × 10^−2^	6.01 × 10^−4^	5.27 × 10^−5^	2.81 × 10^−6^
50#	4.55 × 10^2^	18.3	2.82 × 10^−2^	6.86 × 10^−4^	6.21 × 10^−5^	2.92 × 10^−6^

* The kinetic constants were calculated using the ForteBio’s Octet System. K_on_, association rate con stant; K_dis_, dissociation rate constant; K_D_, dissociation equilibrium constant.

## Data Availability

Not applicable.

## Supplementary Materials

The following supporting information can be downloaded at: https://www.mdpi.com/article/10.3390/ijms232416011/s1, Figure S1: The inhibitory activity of hits against Cathepsin L in vitro; Figure S2: Binding of hits to Norovirus RdRp.
