# Supplementary material for: Identification of Darunavir Derivatives for Inhibition of SARS-CoV-2 3CLpro
Source: Int J Mol Sci. 2022 Dec 16;23(24):16011. doi: 10.3390/ijms232416011 (PMC9781983; doi:10.3390/ijms232416011)
Supplement: Supplementary file 1 [file ijms-23-16011-s001.zip › ijms-1982086-supplementary.pdf]

Identification of Darunavir derivatives for inhibition of SARS-CoV-2 3CLpro

Ling Ma#, Yongli Xie#, Mei zhu, Dongrong Yi, Jianyuan Zhao, Saisai Guo, Yongxin Zhang, Jing Wang, Quanjie Li, Yucheng Wang* and Shan Cen*

[a]*Institute of Medicinal Biotechnology, Chinese Academy of Medical Science and Peking Union Medical College, Beijing 100050, China*

Table of Contents

I.The inhibitory activity of hits against Cathepsin L *in vitro* 2

II.Binding of hits to Norovirus RdRp 2

*Corresponding Authors
E-mail addresses: shancen@imb.pumc.edu.cn. (Shan Cen), wangyucheng@imb.pumc.edu.cn. (Yucheng Wang).
#These authors made equal contributions to this work.

I. The inhibitory activity of hits against Cathepsin L *in vitro*

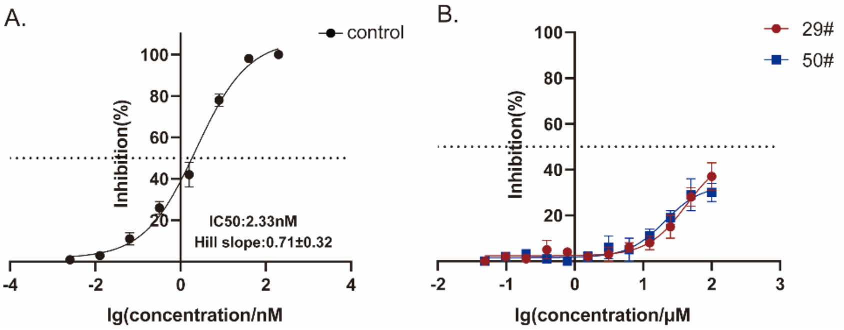

Figure S1. Dose-response inhibition curves of selected hits against Cathepsin L. The calculated initial velocity with each compound was normalized to DMSO control. The results are average ±standard deviation of three repeats.

II. Binding of hits to Norovirus RdRp

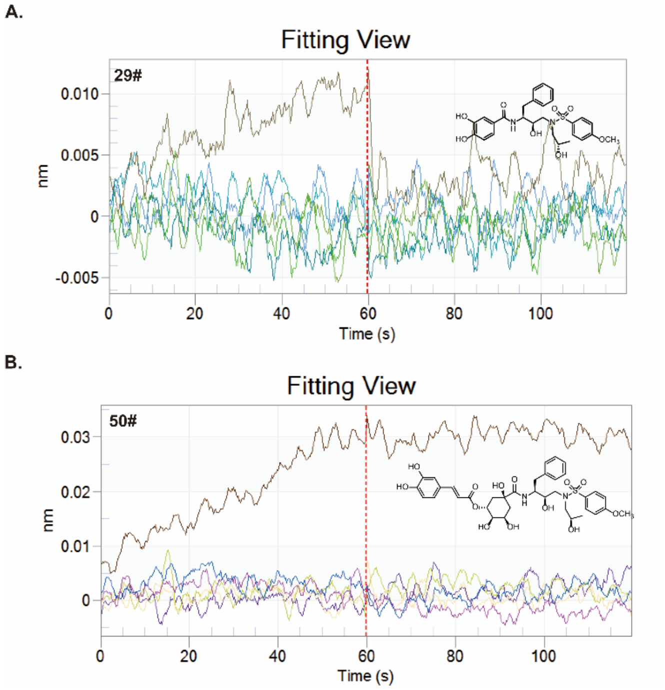

Figure S2. BLI assays for the binding of the hits to Norovirus RdRp protein in vitro. Both of the hits at different serially diluted concentrations were titrated into a fixed concentration of labeled Norovirus RdRp protein.
